# Proteomics analysis reveals heat shock proteins involved in caprine parainfluenza virus type 3 infection

**DOI:** 10.1186/s12917-019-1897-6

**Published:** 2019-05-17

**Authors:** Chunyan Zhong, Jizong Li, Li Mao, Maojun Liu, Xing Zhu, Wenliang Li, Min Sun, Xinqin Ji, Fang Xiao, Leilei Yang, Wenwen Zhang, Zheng Liao

**Affiliations:** 1 Institute of Veterinary Medicine, Jiangsu Academy of Agricultural Sciences, Key Laboratory of Veterinary Biological Engineering and Technology, Ministry of Agriculture, Nanjing, 210014 China; 2 School of Pharmacy, Linyi University, Linyi, 276000 China; 3 College of Animal Science, Guizhou University, Guiyang, 550025 China; 4 Key Lab of Food Quality and Safety of Jiangsu Province-State Key Laboratory Breeding Base, Nanjing, 210014 China

**Keywords:** Caprine parainfluenza virus type 3, Madin-Darby bovine kidney cells, Proteomic analysis, iTRAQ, LC-MS/MS

## Abstract

**Background:**

Caprine parainfluenza virus type 3 (CPIV3) is major pathogen of goat herds causing serious respiratory tract disease and economic losses to the goat industry in China. We analyzed the differential proteomics of CPIV3-infected Madin-Darby bovine kidney (MDBK) cells using quantitative iTRAQ coupled LC-MS/MS. In addition, four DEPs were validated by qRT-PCR and western blot analysis.

**Results:**

Quantitative proteomics analysis revealed 163 differentially expressed proteins (DEPs) between CPIV3-infected and mock-infected groups (*p*-value < 0.05 and fold change > 1.2), among which 91 were down-regulated and 72 were up-regulated. Gene ontology (GO) analysis showed that these DEPs were involved in molecular functions, cellular components and biological processes. Biological functions in which the DEPs were involved in included diseases, genetic information processing, metabolism, environmental information processing, cellular processes, and organismal systems. STRING analysis revealed that four heat shock proteins (HSPs) included HSPA5, HSPA1B, HSP90B1 and HSPA6 may be associated with proliferation of CPIV3 in MDBK cells. qRT-PCR and western blot analysis showed that the selected HSPs were identical to the quantitative proteomics data.

**Conclusion:**

To our knowledge, this is the first report of the proteomic changes in MDBK cells after CPIV3 infection.

**Electronic supplementary material:**

The online version of this article (10.1186/s12917-019-1897-6) contains supplementary material, which is available to authorized users.

## Background

In August 2013, an outbreak of severe goat respiratory disease occurred throughout the major goat herd regions of eastern China. The causative agent was identified as a novel strain of parainfluenza virus type 3 (PIV3) and was designated as caprine parainfluenza virus type 3 (CPIV3) strain JS2013 [1]. The infected goats exhibited high fever, coughing, nasal discharge and dyspnea. Necropsy of the infected goats showed mild to moderate gross lesions in the lungs, and increased amounts of secretion in the tracheas and bronchia were also observed. Genome sequence alignment and phylogenetic analysis revealed that the genome of CPIV3 strain JS2013 showed only 73.3–75.5% identity with BPIV3 and HPIV3 strains [2]. Based on phylogenetic analysis, this pathogen was designated as CPIV3, a member of the PIV3 group belonging to the *Respirovirus* genus within the *Paramyxiviridae* family. Moreover, we further demonstrated that CPIV3 strain JS2013 can be transferred horizontally between adjacent pens [3]. Recently, a seroprevalence study using 2919 serum samples in China reported a CPIV3 prevalence of 39.9% in goats [4]. Another study reported that 35% of nasal swabs and serum samples from clinically diseased goats were positive for CPIV3 by quantitative RT-PCR (qRT-PCR) [5]. It is noteworthy that the spread of CPIV3 has caused heavy economic losses in China [6].

To understand the pathogenesis of viral infection, research on virus-host interaction is critical. Virus infection can dramatically affect host cell morphology, transcription and translation patterns, the cytoskeleton, the cell cycle and innate immune responses of the host, the apoptosis pathway, and may also cause inflammation and alter stress responses [7]. Many functional and morphological changes in host cells are associated with significant changes in the patterns of expression of host cells. Therefore, information on proteome changes in the host following CPIV3 infection may be crucial to understand the host response to viral pathogenesis. In recent years, comparative proteomic analysis has emerged as a valuable tool for the establishment of the global host protein profiles in response to virus infection [8]. This technique has been widely used to investigate proteome changes in cow, yak, buffalo, goat and camel milk [9], and peste des petits ruminants virus (PPRV)-infected Vero cells [10], based on the isobaric tags for relative and absolute quantification (iTRAQ) method. In addition, this technique has also been widely employed to examine the mechanisms of viral infection through comparative investigation of the proteome changes, for example, in the case of Crimean-Congo hemorrhagic fever virus (CCHFV) [11] and bovine respiratory syncytial viruses (BRSV) [12].

However, to the best of our knowledge, no previous study has analyzed the proteomic changes in CPIV3-infected MDBK cells. Proteomic techniques are effective tools to characterize protein expression profiles, and have been widely used to investigate disease-associated proteins [13, 14]. Among current proteomics methods, quantitative high-throughput proteomics approaches are useful for the analysis of infection-associated proteins [15, 16]. In our current study, we used a quantitative proteomics approach based on an iTRAQ tandem mass spectrometry (MS/MS) technique to identify differentially expressed proteins (DEPs) between CPIV3-infected and mock-infected MDBK cells. The functions of the DEPs were analyzed to determine whether they might be associated with CPIV3 infection [17]. Our findings provide valuable insight into the changes in cellular processes that occur during CPIV3 infection.

## Results

### CPIV3 propagation in MDBK cells

**Fig. 1:**
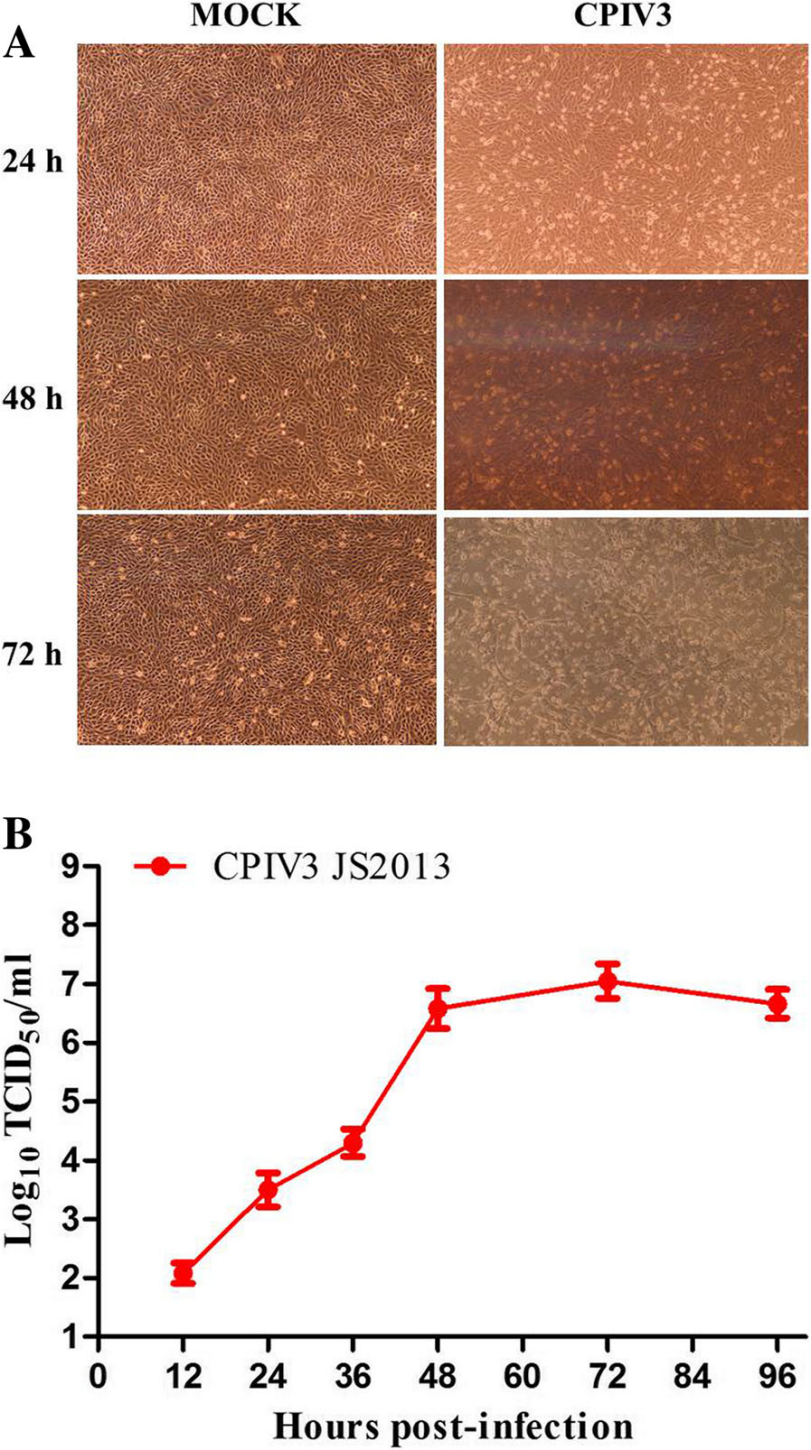
Confirmation of CPIV3 infection in MDBK cells. **a** A CPE was observed in MDBK cells at 24, 48 and 72 h after CPIV3 infection (MOI = 1), with mock-infected cells included as a control. **b** One-step growth curve of CPIV3 strain JS2013 in MDBK cells

The kinetics of CPIV3 propagation in MDBK cells were observed by monitoring the CPE at 24, 48 and 72 h post infection (hpi) (Fig. 1a), a minimal CPE was visible at 24 hpi, whereas an obvious CPE was observed at 48 hpi, and at 72hpi, almost all cells were disrupted. The TCID_50_ showed that the viral titer reached 10^3.5^ TCID_50_/ml at 24 hpi, peaked at 10^7.0^ TCID_50_/ml at 72 hpi and then declined (Fig. 1b)**.** To ensure a higher proportion of infected cells and to avoid an excessive CPE, we selected 24 hpi as the time point under our infection conditions for further proteomic analysis.

### Identification and annotation of proteins

We detected 8153 proteins and quantified 4109 proteins, including 28,815 peptides (Additional file 1: Figure S1). Detected proteins were annotated according to the GO database in the following categories: cellular components (CC), biological processes (BP), and molecular functions (MF) (Additional file 2: Figure S2). The top 20 pathways containing the largest number of proteins among the 8153 proteins were annotated according to KEGG (Additional file 3: Figure S3). Based on the KOG, 830 of the proteins were annotated as being involved in information storage and processing, 1545 were annotated as cellular processes and signaling, 581 were annotated as metabolism, and 699 were annotated as poorly characterized (Additional file 4: Figure S4 and Data Sheet 5). Furthermore, the cutoff criteria considered for the DEPs were set with an adjusted *p-*value of < 0.05 and a ratio of > 1.2-fold difference. Among the DEPs, 163 proteins from the two sets of biological replicates overlapped and were subsequently adjusted for multiple testing according to the stringent method of Benjamini and Hochberg [18]. Of these, 72 proteins were up-regulated and 91 proteins were down-regulated based on our criteria for the identification of DEPs in the MDBK-infected and mock-infected groups using the iTRAQ-MS/MS approach. Protein ratios were presented as CPIV3-infected/mock-infected. An average V/C ratio > 1 represented up-regulated proteins and an average V/C ratio < 1 represented down-regulated proteins. A list of DEPs information is shown in Table 1. DEPs between the two groups are shown as heat map and scatterplot (Additional file 5: Figure S6 and S7). Finally, the DEPs displaying the greatest increase and decrease in expression in the CPIV3-infected MDBK cells were FAM81B protein (1:0.118) and the DEP displaying the greatest decrease in expression in the CPIV3-infected MDBK cells was carboxypeptidase (1:1.206).

**Table 1:** Statistically significant DEPs identified by iTRAQ analysis of MDBK cells infected with CPIV3

Accession	Protein name	CPIV3-infected	Mock-infected	FC (CPIV3-infected vs_Mock-infected)	regulate
Q0VCX2	Endoplasmic reticulum chaperone BiP (HSPA5)	1	0.488	2.049180328	up
Q95M18	Endoplasmin (HSP90B1)	1	0.744	1.344086022	up
F1MEN8	Protein disulfide-isomerase A4 (PDIA4)	1	0.816	1.225490196	up
E1B748	Hypoxia up-regulated protein 1 precursor (HYOU1)	1	0.704	1.420454545	up
Q27965	Heat shock 70 kDa protein 1B (HSPA1B)	1	0.793	1.261034048	up
A6QR28	Phosphoserine aminotransferase (PSAT1)	1	0.814	1.228501229	up
F1MWU9	Uncharacterized protein (HSPA6)	1	0.726	1.377410468	up
Q3ZCA7	G protein subunit alpha i3 (GNAI3)	1	0.739	1.353179973	up
Q1LZA3	Asparagine synthetase [glutamine-hydrolyzing] (ASNS)	1	0.721	1.386962552	up
P80513	Mesencephalic astrocyte-derived neurotrophic factor (MANF)	1	0.794	1.259445844	up
Q2KHU0	Phosphoserine phosphatase (PSPH)	1	0.81	1.234567901	up
Q3T0L2	Endoplasmic reticulum resident protein 44 (ERP44)	1	0.765	1.307189542	up
Q08DL0	SLC3A2 protein (SLC3A2)	1	0.78	1.282051282	up
A5PK96	ACP1 protein (ACP1)	1	0.78	1.282051282	up
P13909	Plasminogen activator inhibitor 1 (SERPINE1)	1	0.807	1.239157373	up
P68301	Metallothionein-2 (MT2)	1	0.491	2.036659878	up
Q27955	Voltage-gated potassium channel subunit beta-2 (KCNAB2)	1	0.797	1.254705144	up
A5D7C1	Probable ATP-dependent RNA helicase DDX52 (DDX52)	1	0.814	1.228501229	up
A6H797	MLEC protein (MLEC)	1	0.807	1.239157373	up
F1N1R3	Mitochondrial ribosomal protein L40 (MRPL40)	1	0.824	1.213592233	up
E1BPL3	ATP binding cassette subfamily B member 7 (ABCB7)	1	0.721	1.386962552	up
A5PJN8	Splicing factor 3A subunit 2 (SF3A2)	1	0.551	1.814882033	up
Q2KIN6	Protein Mpv17 (MPV17)	1	0.801	1.248439451	up
Q3SZZ0	Ribosome biogenesis protein BRX1 homolog (BRIX1)	1	0.799	1.251564456	up
A6QLR4	Flotillin-2 (FLOT2)	1	0.476	2.100840336	up
Q17QI2	RNA polymerase II subunit A C-terminal domain phosphatase SSU72 (SSU72)	1	0.796	1.256281407	up
Q0VCS9	Ankyrin repeat and MYND domain-containing protein 2 (ANKMY2)	1	0.812	1.231527094	up
A2VE10	Protein CASC4 (CASC4)	1	0.829	1.206272618	up
A7MB19	NLRX1 protein (NLRX1)	1	0.804	1.243781095	up
Q6EVI2	eIF4GI protein (eIF4GI)	1	0.825	1.212121212	up
Q3SZ99	Peptidylprolyl isomerase (AIP)	1	0.774	1.291989664	up
E1BD11	Chromosome 11 open reading frame 84 (SPINDOC)	1	0.825	1.212121212	up
A4FUC0	39S ribosomal protein L37, mitochondrial (MRPL37)	1	0.815	1.226993865	up
Q2TA30	Ninjurin 1 (NINJ1)	1	0.594	1.683501684	up
E1BN60	Solute carrier family 30 member 1 (SLC30A1)	1	0.77	1.298701299	up
Q3T093	Adaptin ear-binding coat-associated protein 1 (NECAP1)	1	0.83	1.204819277	up
G3N3D6	Phosphoinositide phospholipase C(PLCH1)	1	0.823	1.215066829	up
Q2YDF6	28S ribosomal protein S35, mitochondrial(MRPS35)	1	0.809	1.236093943	up
Q08DH9	CCCTC-binding factor(CTCF)	1	0.802	1.246882793	up
Q08DK7	Mitochondrial basic amino acids transporter(SLC25A29)	1	0.798	1.253132832	up
F1MBD5	Surfeit 2(SURF2)	1	0.833	1.200480192	up
G3X6N3	Serotransferrin (TF)	1	0.76	1.315789474	up
F1MG47	Peroxisomal N(1)-acetyl-spermine/spermidine oxidase (PAOX)	1	0.706	1.416430595	up
E1BH45	RB1 inducible coiled-coil 1 (RB1CC1)	1	0.682	1.46627566	up
E1BMF4	Kinase D interacting substrate 220 (KIDINS220)	1	0.811	1.233045623	up
E1BI11	ELM2 and Myb/SANT domain containing 1 (ELMSAN1)	1	0.736	1.358695652	up
Q5E9T1	GDP-D-glucose phosphorylase 1 (GDPGP1)	1	0.823	1.215066829	up
A7Z023	CCDC132 protein (CCDC132)	1	0.819	1.221001221	up
A6QR26	UBAP1 protein (UBAP1)	1	0.702	1.424501425	up
A5PJZ7	Histone deacetylase (HDAC6)	1	0.832	1.201923077	up
Q148F0	Ubiquitin-related modifier 1 (URM1)	1	0.402	2.487562189	up
F1MRI6	Lemur tyrosine kinase 2 (LMTK2)	1	0.42	2.380952381	up
Q0V882	Bax inhibitor 1 (TMBIM6)	1	0.766	1.305483029	up
G3X6Y2	Chromosome X open reading frame 38 (CXHXorf38)	1	0.81	1.234567901	up
G3MYB9	UNC homeobox (UNCX)	1	0.793	1.261034048	up
G3N0M5	Uncharacterized protein	1	0.698	1.432664756	up
Q3SZN3	Metalloendopeptidase OMA1, mitochondrial (OMA1)	1	0.304	3.289473684	up
A7YWG9	PHLDA1 protein (PHLDA1)	1	0.654	1.529051988	up
A0JNQ0	Allograft inflammatory factor 1-like (AIF1L)	1	0.808	1.237623762	up
Q2YDD1	FGFR1 oncogene partner (FGFR1OP)	1	0.664	1.506024096	up
F1MN39	Interferon related developmental regulator 1 (IFRD1)	1	0.521	1.919385797	up
Q0II90	Protein FAM81B (FAM81B)	1	0.118	8.474576271	up
Q75V95	Calcitonin receptor-stimulating peptide 1 (CRSP1)	1	0.818	1.222493888	up
F1MSI9	Discs large MAGUK scaffold protein 5 (DLG5)	1	0.795	1.257861635	up
E1BFR6	Transmembrane protease, serine 13 (TMPRSS13)	1	0.827	1.209189843	up
E1BC24	Midasin (MDN1)	1	0.579	1.727115717	up
Q08DG0	Nuclear receptor binding factor 2 (NRBF2)	1	0.434	2.304147465	up
Q2KI89	LisH domain-containing protein ARMC9 (ARMC9)	1	0.411	2.433090024	up
F1MNN5	Sortilin related VPS10 domain containing receptor 1 (SORCS1)	1	0.759	1.317523057	up
A0A140T882	Uncharacterized protein CLBA1 (CLBA1)	1	0.635	1.57480315	up
F1MH73	Transmembrane protein 131 (TMEM131)	1	0.793	1.261034048	up
Q28037	Vitamin D3 receptor (VDR)	1	0.826	1.210653753	up
F1N2K8	Periplakin (PPL)	1	1.423	0.702740689	down
F6RJG0	3-hydroxy-3-methylglutaryl coenzyme A synthase (HMGCS1)	1	1.371	0.729394602	down
Q5KR49	Tropomyosin alpha-1 chain (TPM1)	1	1.227	0.814995925	down
G3MWV5	Histone cluster 1 H1 family member e (HIST1H1E)	1	1.264	0.791139241	down
A7MAZ5	Histone H1.3 (HIST1H1D)	1	1.258	0.79491256	down
Q3SYV6	Importin subunit alpha (KPNA2)	1	1.22	0.819672131	down
Q28178	Thrombospondin-1 (THBS1)	1	1.406	0.711237553	down
F1N3A1	Thrombospondin-1 (THBS1)	1	1.555	0.643086817	down
A4FV94	KRT6A protein (KRT6A)	1	1.212	0.825082508	down
A6QPB5	PGM1 protein (PGM1)	1	1.272	0.786163522	down
G3N0V2	Keratin 1 (KRT1)	1	1.499	0.667111408	down
E1BNE7	Caveolae associated protein 1 (CAVIN1)	1	1.213	0.824402308	down
Q3YJF3	MHC class I antigen (Fragment) (BoLA)	1	1.277	0.783085356	down
Q2HJJ0	Kinesin light chain 4 (KLC4)	1	1.209	0.827129859	down
F1MX88	Solute carrier family 25 member 13 (SLC25A13)	1	1.212	0.825082508	down
F1 N688	V-type proton ATPase subunit B, kidney isoform (ATP6V1B1)	1	1.352	0.73964497	down
Q0VCZ8	Acyl-CoA synthetase long-chain family member 1 (ACSL1)	1	1.333	0.750187547	down
A6QNZ7	Keratin 10 (Epidermolytic hyperkeratosis; keratosis palmaris et plantaris) (KRT10)	1	1.387	0.720980534	down
F1N4K3	Uncharacterized protein	1	1.474	0.678426052	down
F1MTJ9	Terpene cyclase/mutase family member (LSS)	1	1.243	0.804505229	down
Q867D1	Stearoyl-CoA desaturase (Scd)	1	1.427	0.700770848	down
F1MH31	Nucleoporin 214 (NUP214)	1	1.241	0.805801773	down
G3N1R5	Uncharacterized protein	1	1.454	0.687757909	down
Q32PA5	Ubiquitin-conjugating enzyme E2 C (UBE2C)	1	1.589	0.629326621	down
Q0P5J6	Keratin, type I cytoskeletal 27 (KRT27)	1	1.375	0.727272727	down
A7MB38	SFRS4 protein (SRSF4)	1	1.22	0.819672131	down
A7YW33	DNA polymerase delta interacting protein 3 (POLDIP3)	1	1.267	0.789265983	down
Q3ZCI0	Coiled-coil-helix-coiled-coil-helix domain containing 2 (CHCHD9)	1	1.298	0.770416025	down
E1BJC9	Uncharacterized protein (C18H19orf33)	1	1.24	0.806451613	down
A5D7N6	Kinesin-like protein (KIF23)	1	1.373	0.728332119	down
F2Z4H2	Non-histone chromosomal protein HMG-17 (HMGN2)	1	1.242	0.805152979	down
A3KLR9	Superoxide dismutase (SOD3)	1	1.36	0.735294118	down
G8JKY5	Thymosin beta-4 (TMSB4X)	1	1.547	0.646412411	down
Q08DI5	Ras-related protein Rap-2c (RAP2C)	1	1.207	0.828500414	down
A4IF70	GPR56 protein (GPR56)	1	1.233	0.811030008	down
P15103	Glutamine synthetase (GLUL)	1	1.265	0.790513834	down
E1BKT0	Leucine zipper protein 1 (LUZP1)	1	1.353	0.7390983	down
F1MFW9	Keratin 24 (KRT24)	1	2.313	0.432338954	down
Q0VC74	Trimethyllysine dioxygenase, mitochondrial (TMLHE)	1	1.217	0.821692687	down
F1MLZ1	Cytochrome b reductase 1 (CYBRD1)	1	1.252	0.798722045	down
F1MP14	Forkhead box K1 (FOXK1)	1	1.208	0.82781457	down
F1MYS2	FCH domain only 2 (FCHO2)	1	1.253	0.798084597	down
Q3T0J9	Guanine nucleotide-binding protein-like 3-like protein (GNL3L)	1	1.336	0.748502994	down
Q2NKZ9	Carboxypeptidase (SCPEP1)	1	1.206	0.829187396	down
F1N6L1	Valyl-tRNA synthetase 2, mitochondrial (VARS2)	1	1.272	0.786163522	down
G5E5Q8	SET binding factor 1 (SBF1)	1	1.286	0.777604977	down
Q2KHW7	Regulator of G-protein signaling 10 (RGS10)	1	1.219	0.820344545	down
F1N4R2	Uncharacterized protein (MORF4L1)	1	1.222	0.818330606	down
Q5E9Q1	Protein O-glucosyltransferase 1 (POGLUT1)	1	1.234	0.810372771	down
Q29RZ9	WD repeat-containing protein 92 (WDR92)	1	1.26	0.793650794	down
F1N5R4	Conserved oligomeric Golgi complex subunit 8 (COG8)	1	1.271	0.786782061	down
F1ML71	Nedd4 family interacting protein 2 (NDFIP2)	1	1.254	0.797448166	down
G3 N266	G protein signaling modulator 1 (GPSM1)	1	1.235	0.809716599	down
F1N0K0	Collagen alpha-1(XI) chain (COL11A1)	1	1.212	0.825082508	down
F1MGF2	Chromodomain helicase DNA binding protein 1 (CHD1)	1	1.254	0.797448166	down
A6QQK2	MAP3K7IP1 protein (MAP3K7IP1)	1	1.339	0.74682599	down
E1BDA1	Ras and Rab interactor 1 (RIN1)	1	1.29	0.775193798	down
E1B8R7	HPS5, biogenesis of lysosomal organelles complex 2 subunit 2 (HPS5)	1	1.252	0.798722045	down
A8E646	CARD11 protein (CARD11)	1	1.222	0.818330606	down
Q32KL9	B-cell receptor-associated protein 29 (BCAP29)	1	1.435	0.696864111	down
E1BGG6	Regulatory factor X5 (RFX5)	1	1.233	0.811030008	down
Q3T0N3	Calcium load-activated calcium channel (TMCO1)	1	1.295	0.772200772	down
E1BC89	Oxysterol-binding protein (OSBPL5)	1	1.241	0.805801773	down
F1MQ45	Solute carrier organic anion transporter family member (SLCO2A1)	1	1.234	0.810372771	down
Q32P76	Small EDRK-rich factor 1 (SERF1)	1	1.447	0.691085003	down
A6QQS5	WHSC2 protein (WHSC2)	1	1.289	0.77579519	down
F1MNT2	Protein RTF2 homolog (RTF2)	1	1.278	0.782472613	down
F1MEY2	Enoyl-[acyl-carrier-protein] reductase, mitochondrial (MECR)	1	1.387	0.720980534	down
A6QNX2	DPP7 protein (DPP7)	1	1.287	0.777000777	down
E1BE80	Transmembrane protein 236 (TMEM236)	1	1.247	0.801924619	down
A4IFD1	PDCD4 protein (PDCD4)	1	1.209	0.827129859	down
A1A4R8	Cell division cycle protein 23 homolog (CDC23)	1	1.267	0.789265983	down
E1BG49	Centromere protein E (CENPE)	1	1.324	0.755287009	down
P07926	ATP synthase F(0) complex subunit C2, mitochondrial (ATP5MC2)	1	1.497	0.668002672	down
Q402A0	Aggrus (PDPN)	1	1.293	0.773395205	down
Q17QI1	Trafficking protein particle complex subunit 1 (TRAPPC1)	1	1.265	0.790513834	down
E1BKA4	Uncharacterized protein (HAUS4)	1	1.3	0.769230769	down
Q2KHT6	F-box only protein 32 (FBXO32)	1	1.227	0.814995925	down
F1MS44	Doublecortin domain containing 2 (DCDC2)	1	1.277	0.783085356	down
E1BIR2	Dipeptidase (DPEP2)	1	1.211	0.825763832	down
A5PKA5	Sorting nexin-27 (SNX27)	1	1.31	0.763358779	down
A6H7C1	MORF4L2 protein (MORF4L2)	1	1.213	0.824402308	down
A6QLZ5	Protein FAM177A1 (FAM177A1)	1	1.23	0.81300813	down
P13384	Insulin-like growth factor-binding protein 2 (IGFBP2)	1	1.748	0.57208238	down
A5D974	Acyl-Coenzyme A dehydrogenase family, member 9 (ACAD9)	1	1.217	0.821692687	down
F1N2N9	Coiled-coil domain containing 114 (CCDC114)	1	1.224	0.816993464	down
E1BBH4	Protein unc-93 homolog B1 (UNC93B1)	1	1.666	0.600240096	down
A5PJX0	F-box protein 22 (FBXO22)	1	1.272	0.786163522	down
E1BEG4	Zinc finger FYVE-type containing 16 (ZFYVE16)	1	1.225	0.816326531	down
E1BEI6	ATM serine/threonine kinase (ATM)	1	1.507	0.663570007	down
P0C914	Overexpressed in colon carcinoma 1 protein homolog	1	1.213	0.824402308	down

### GO analysis of the DEPs

**Fig. 2:**
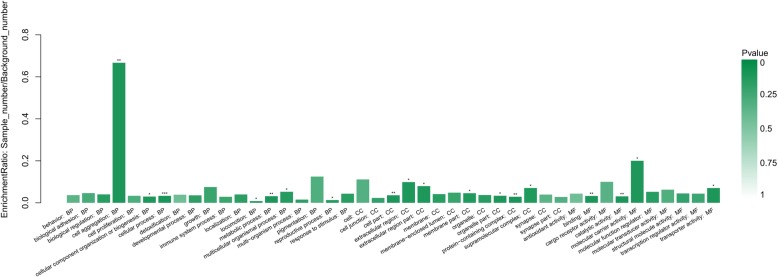
GO enriched histogram of DEPs. Each column in the figure is a GO terms, the abscissa text indicates the name and classification of GO, and the height of the column indicates the enrichment rate. The color indicates the significance of the enrichment (*p*-value). The darker the color, the more significant the enrichment of the GO term (*P < 0.05; **P < 0.01; ***P < 0.001)

The molecular functional classes and subcellular locations of the 163 DEPs were analyzed using UniProt and the GO database. The 163 DEPs were annotated into the categories: cellular component, biological process, or molecular function, and the distribution of up-regulated and down-regulated proteins among these GO annotations are shown in Additional file 6: Figure S8. GO enrichment annotation comparisons were performed to elucidate the characteristics of the altered proteins in MDBK cells induced by CPIV3 infection, to determine any associations with virulence and pathogenicity. In terms of biological process annotation, DEPs were mainly involved in cell aggregation, cellular processes, cellular component organization or biogenesis, locomotion, metabolic processes, multicellular organismal processes and reproductive processes; in terms cellular component annotation, DEPs were mainly involved in the cell part, extracellular region part, membrane part, organelle part, protein-containing complex and supramolecular complex; in terms of molecular function annotation, DEPs were mainly involved in binding, catalytic activity, molecular carrier activity and transporter activity (Fig. 2).

### KEGG (Kyoto encyclopedia of genes and genomes) pathway analysis of the DEPs

**Fig. 3:**
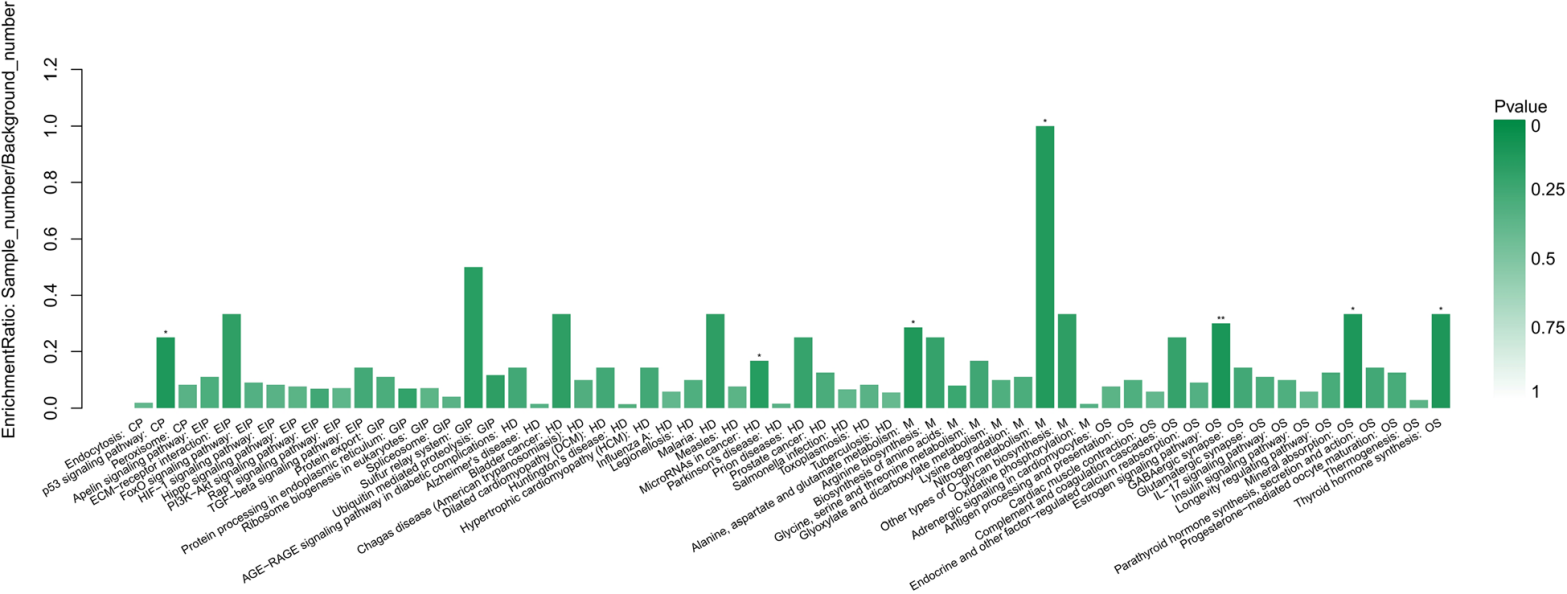
KEGG enrichment annotation of the DEPs. Each column in the figure is a pathway. The abscissa text indicates the name and classification of the pathway, and the height of the column indicates the enrichment rate. The color indicates the significance of the enrichment (*p*-value). The darker the color, the more significant the enrichment of the pathway (*P < 0.05; **P < 0.01; ***P < 0.001)

**Fig. 4:**
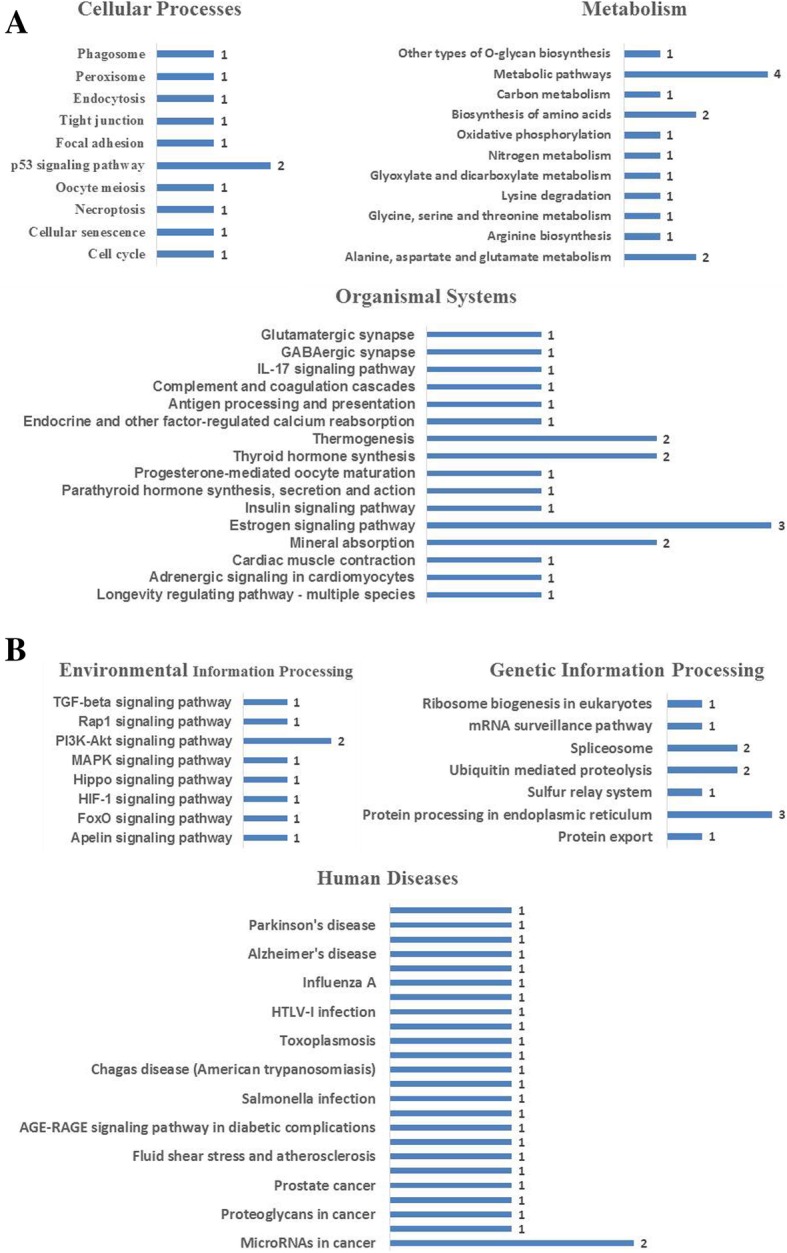
Functional characterization of DEPs. **a** Cellular processes, metabolism and organismal systems. **b** Environmental information processing, genetic information processing and human diseases. More information is available in Additional file 5: Figure S7

The KEGG pathway is a collection of pathway maps that represent molecular interactions and reaction networks in cell line. The 93 DEPs identified were annotated, and mapped to a total of six KEGG pathway categories, which included metabolism, disease, genetic information processing, cellular processes, environmental information processing, and organismal systems pathway categories (Additional file 7 Data Sheet 9). The enrichment annotation protein pathway information is shown in Fig. 3. The results showed that most of the abundant KEGG terms were involved in biological processes such as the p53 signaling pathway, microRNAs in cancer, alanine, aspartate and glutamate metabolism, nitrogen metabolism, the estrogen signaling pathway, mineral absorption and thyroid hormone synthesis. Functional classification by KEGG showed that the upregulated and downregulated proteins could be divided among six distinct functional sets: environmental information processing, cellular processes, metabolism, genetic information processing, organismal systems and human diseases (Fig. 4).

### STRING analysis of the relationships between DEPs

**Fig. 5:**
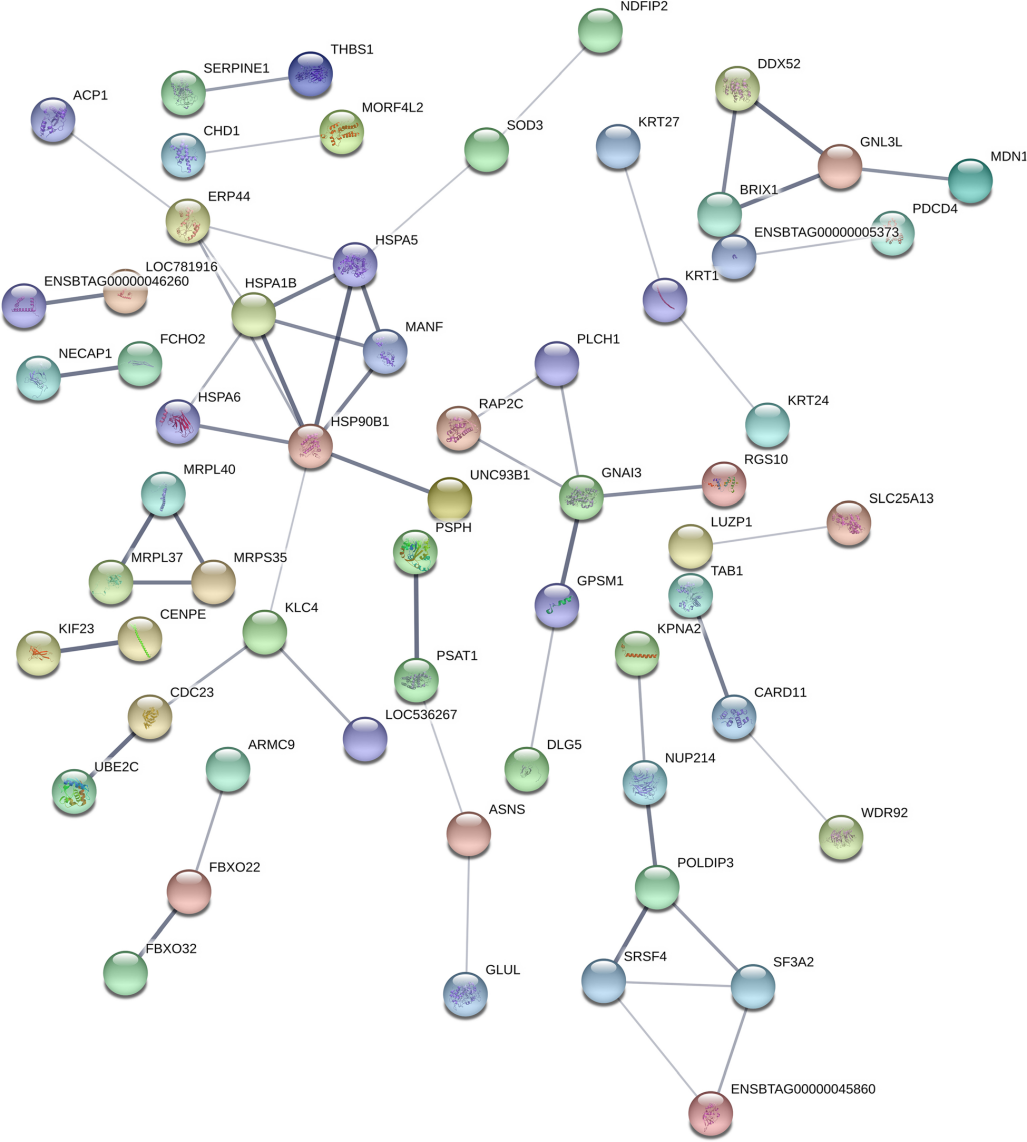
Specific network analysis of proteins significantly altered in CPIV3-infected cells. The network of DEPs with STRING analysis. Each node represents a protein in the graph, each line represents the interaction between proteins, and the wider the line, the closer the relationship

With the goal of exploring the potential protein network connections for the differentially regulated proteins in detail, the STRING tool was used. The differentially regulated proteins were mainly mapped to four functional networks (Fig. 5). A specific network had at least four “focus” proteins (HSPA5, HSPA1B, HSP90B1 and HSPA6). The networks of interest corresponded to: cell-to-cell signaling, hereditary disorder, cell death and survival, cardiovascular disease, cellular developmental, RNA post-transcriptional modification, cellular growth and proliferation.

### Confirmation of proteomic data by qRT-PCR

**Fig. 6:**
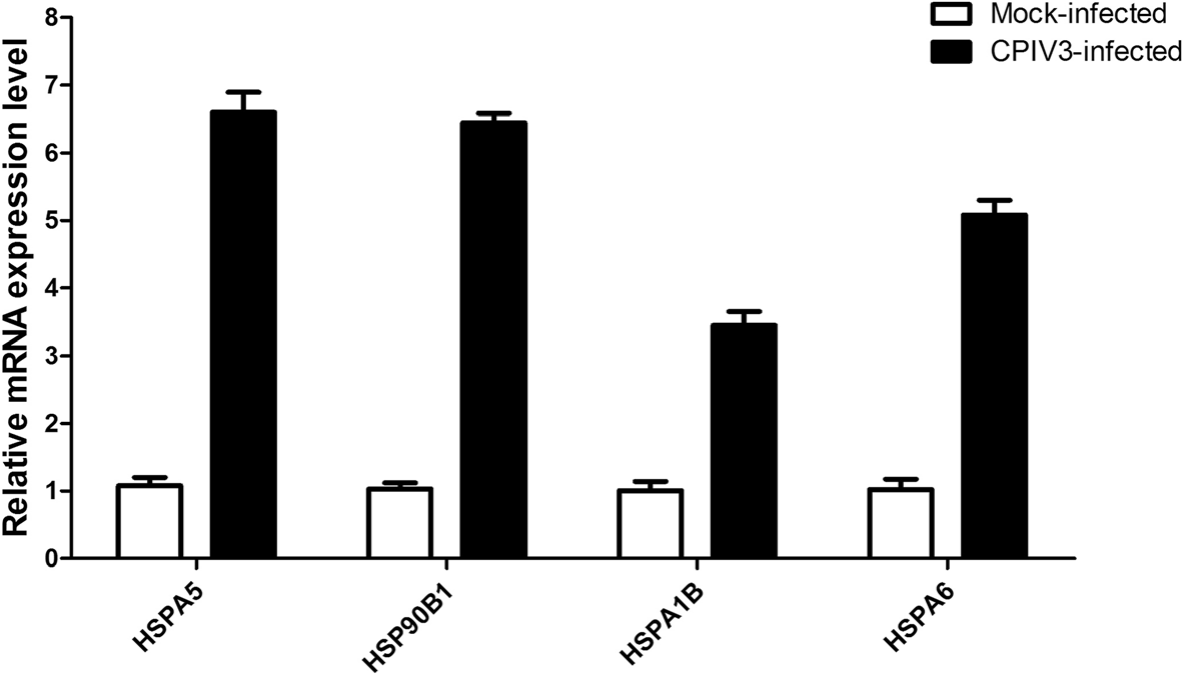
qRT-PCR analysis of mRNA expression in the CPIV3-infected and mock-infected groups. The cells were collected at 24 hpi for qRT-PCR to analyze the relative mRNA expression of the HSPA5, HSP90B1, HSPA1B and HSPA6 genes. The GAPDH gene was included as a control housekeeping gene for the normalization of samples . Error bars represent standard deviations

Alterations in the expression of a protein may be owing to a change in its mRNA levels. To confirm the results of the proteomic analysis by mRNA expression, transcriptional alterations in four selected proteins were measured by qRT-PCR. The qRT-PCR analysis showed that no difference in the ratio of these mRNAs between the CPIV3 infected group and the mock infected group were consistent with those obtained using quantitative proteomics analysis (Fig. 6). The mRNA expression of HSPA5, HSP90B1, HSPA1B and HSPA6 were increased in CPIV3-infected MDBK cells. Therefore, the trends in the mRNA expression were consistent with those in their corresponding proteins.

### Western blot analysis of HSPA1B

**Fig. 7:**
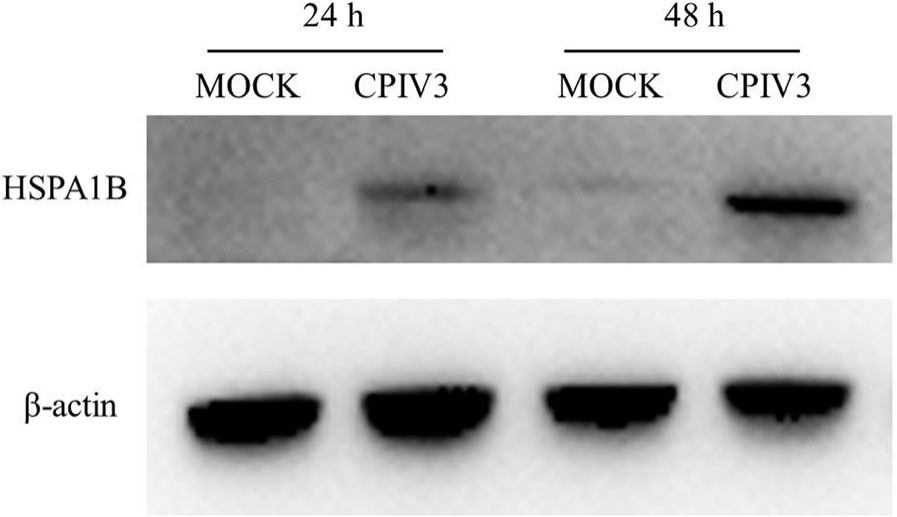
Analysis of HSPA1B expression levels in CPIV3-infected and control cells by western blot analysis at 24 h and 48 h. Protein samples were separated by SDS-PAGE. Western blot analysis was performed using antibodies to the HSPA1B protein.β-actin protein was detected as an internal control

We analyzed the expression levels of HSPA1B (up-regulated) in CPIV3-infected MDBK cells (Fig. 7) by western blot at 24 h and 48 h. Figure 7 shows that HSPA1B was up-regulated in CPIV3-infected MDBK cells at 24 h and 48 h. The results were consistent with those obtained using the iTRAQ labeled LC-MS/MS system.

## Discussion

Proteomic techniques have become significant methodologies for determining cellular protein interactions and host cellular pathophysiological processes following virus infection [19, 20]. As a general rule, no important host cell membrane rearrangement or cytoskeleton collapse is observed following virus infection but the point at which a high virus yield is obtained is considered as the best time for proteomic analysis [21, 22]. Taking this substantial evidence into consideration, cell samples at 24 hpi were chosen for further proteomic analysis. Based on our study, the expression levels of 163 DEPs were found to be significantly altered in CPIV3-infected cells. The results of GO, KEGG pathway and STRING analysis predicted that these DEPs pertaining to different types of functional categories and signal pathways. Western blot and qRT-PCR were also applied to validate some differential proteins at the mRNA and protein levels. To date, no analysis has been reported of the differential proteomes of MDBK cells infected with CPIV3. Our data may provide an overview of the proteins altered in expression during the host response to CPIV3 infection and may provide insight in the process of CPIV3 pathogenesis.

Studies have shown that HSPs may play an important role in virus host cell interactions during in vivo and in vitro infection [23, 24]. Inhibitors of HSP90 can inhibit herpes simplex virus type 1 (HSV-1) infection [25]. Bovine viral diarrhea virus (BVDV) structural proteins comprise the C nucleocapsid protein and three envelope glycoproteins, Erns, E1 and E2 [26]. A previous study found that HSP110 enhanced the presentation of E2 to CD4 T cells in vitro to improve the immunogenicity of an E2 vaccine in cattle [27]. Previous work demonstrated that HSP70 is actively released into the extracellular milieu and acts as a cytokine and peptide adjuvant, thereby promoting both the innate and adaptive immune responses [28]. In our analysis, four proteins (HSPA5, HSPA1B, HSP90B1 and HSPA6) were identified following CPIV3 infection. HSP90B1 is proposed to be associated with poor survival from hepatocellular carcinoma (HCC), whereas high levels of HSPA5 and HSPA6 may be associated with earlier recurrence of HCC [29]. HSPA1B, also known as heat shock protein 72, is a member of the HSP70 family. HSP70 expression levels rapidly increased in response to cellular stresses such as heat shock, or in response to certain viral infections [30–33].

In the current study, HSP70 was rarely detected in the mock-infected group, whereas it was notably present in the CPIV3 group. CPIV3 infection resulted in the up-regulated secretion of exosomes and packaging of the viral proteins into exosomes, and these results suggested that CPIV3 infection may enhance HSP70-mediated exosome release (unpublished data). In addition, HSP70 is actively released into the extracellular milieu, thereby promoting innate and adaptive immune responses [34]. In this study, HSPA5, HSPA1B, HSP90B1 and HSPA6 were up-regulated at 24 hpi to various degrees following CPIV3 -infection of MDBK cells. Different expression levels of HSPA1B were detected by western blot analysis at 24 hpi and 48 hpi after CPIV3 -infection of MDBK cells. This may indicate that HSPA1B affects the proliferation of CPIV3 in MDBK cells. HSPA1B is an endogenous ligand for toll-like receptor TLR4, thereby stimulating innate immunity [35], and HSPA1B regulates the NF-κB pathway via TLR2 and TLR4 in fibroblasts. However, fibroblasts and macrophages interact with each other to mediate the immune response. Activation of the NF-κB pathway then results to in enhanced secretion of pro-inflammatory cytokines (TNF-α, IL-6 and IL-1β) and neutrophil chemoattractant MIP-2 and Cxcl1 from macrophages [36]. This evidence indicates that HSPA1B may be associated with the proliferation of CPIV3 in MDBK cells through an ability to interact with key components of the NF-κB pathway, moreover, those involved in innate immunity, but the detailed mechanism remains unknown. However, the detailed functions of these pathways and proteins changes in CPIV3 infection therefore requires further verification.

## Conclusions

The proteomic changes in CPIV3-infected MDBK cells were analyzed using iTRAQ combined with LC-MS/MS. To the best of our knowledge, this is the first time proteomics has been used to explore the virus–host protein interaction network in CPIV3-infected MDBK cells. The results revealed 163 DEPs, among which 72 were up-regulated and 91 were down-regulated. In addition, four DEPs were validated by qRT-PCR and HSPA1B was validated by western blot analysis. These results were consistent with those of label-free LC-MS analysis. Our analyses of the DEPs were descriptive, and further functional investigations are required to elucidate the pathogenic mechanisms and cellular responses to CPIV3 infection.

## Methods

### Cell culture and virus infection

CPIV3 strain JS2013 isolated in Jiangsu Province was used for virus infection. MDBK cells were cultured in Dulbecco’s modified Eagle’s medium (DMEM; Sigma, CA, USA) supplemented with 10% fetal bovine serum (FBS; HyClone, UT, USA), at 37 °C in an atmosphere of 5% CO_2_ [2]. When the cells grow to 70–80% confluence, they were inoculated with CPIV3 at a multiplicity of infection (MOI) of 1. After 1 h of adsorption, infected cells were maintained in fresh medium containing 2% FBS. Uninfected cells were used as a control. The CPIV3- or mock-infected cells were collected at 24 hpi. Viral propagation was confirmed by the observation of a cytopathic effect (CPE).

### Protein sample preparation and labeling with iTRAQ reagent

The CPIV3- and mock-infected cell samples were washed three times with cold phosphate-buffered saline (PBS) and then treated with lysis buffer containing 8 M urea, 4% CHAPS, 2 M thiourea, and 30 mM Tris-HCl on ice for 30 min until the cell line were completely lysed. The supernatant was collected by centrifugation at 12000×*g* for 30 min at 4 °C after ultrasonication treatment for 2 min. The protein concentration in the supernatants was quantified using the Bradford protein assay. After reduction and cysteine-blocking as described in the iTRAQ protocol (AB Sciex, Concord, ON, USA), solutions containing 100 μg protein were digested overnight at 37 °C with sequence grade modified trypsin (Promega, Madison, WI, USA) and then labeled with different iTRAQ tags. The labeled samples were then mixed and dried with a rotary vacuum concentrator.

### LC-MS analysis

Ten microliters (μl) of each fraction were analyzed by Q Exactive (Thermo, USA) mass spectrometer coupled to a Proxeon Biosystem Easy-nLC 1200 (Thermo Fisher Scientific, Waltham, MA, USA) in the LC-MS experiments. The peptide mixture (5 g) was loaded onto a C18 column (75 μm × 25 cm, Thermo,USA) packed with RP-C18 (5 m) resin in buffer A (2% ACN with 0.1% formic acid), and eluted with a linear gradient of buffer B (80% ACN with 0.1% formic acid) at a flow rate of 300 nl/min for 120 min using IntelliFlow technology. The equate underwent electrospray ionization for LC-MS analysis. The MS/MS instrument was run in the peptide recognition mode, and the spectra were acquired using a data-dependent top-20 method based on the selection of the most abundant precursor ions from the survey scan (350–1300 m/z) for HCD fragmentation. Determination of the target value was based on the predictive automatic gain control, and the dynamic exclusion duration was 18 s. Survey scans were acquired at a resolution of 70,000 at m/z 200, and the resolution for the HCD spectra was set to 17,500 at m/z 200. The normalized collision energy was 30 eV, and the underfill ratio, which specifies the minimum percentage of the target value likely to be reached at maximum fill time, was defined as 0.1%. Thermo Xcalibur 4.0 (Thermo, USA) was used to collect MS analysis data via DDA mode.

### Data analysis

The MS data were analyzed using Proteome Discoverer™ software 2.1. When the library was searched, the raw file was submitted to the Proteome Discoverer server searched against the Uniprot *Bos taurus* database (197,939 total sequences, downloaded April 26, 2018). The following parameters were used for protein identification: a precursor mass tolerance of 20 ppm; a fragment mass tolerance of 0.05 Da; trypsin digestion; max. Missed cleavage sites of 2; the variable dynamic modifications included oxidation (M), iTRAQ8plex (Y) and acetyl (protein N-terminus), and the fixed static modifications included carbamidomethyl (C), iTRAQ8plex (K) and iTRAQ8plex (N-term). The cutoff for the global false discovery rate (FDR) for peptide and protein identification was set to 0.01. The value of the quantitative ratio for each protein relative to the internal reference was calculated, and averaged to obtain the quantitative ratio (V/C) of the proteins identified in the treatment groups [37]. Proteins with a fold change > 1.2 and a *p*-value < 0.05 were considered to shows significantly different expression. Auto bias-correction was executed to decrease the artificial error. Statistical analysis was performed using Excel 2007 software. The DEPs were annotated using gene ontology (GO) and KEGG database. The Cluster of Orthologous Groups of proteins (COG or KOG) were retrieved, and mapped to pathways in the KEGG database [38]. In addition, DEPs were analyzed using STRING for predicting functional association networks of proteins.

### CPIV3 yield quantification

MDBK cells were seeded in 96-well plates and incubated for 24 h. Then, CPIV3 samples were 10-fold serially diluted and added to each well in quadruplicate. MDBK cells exhibit CPE were scored positive for viral growth and the TCID_50_ was calculated by the Reed–Muench method [39].

### mRNA quantitation by qRT-PCR

Total cellular RNA was extracted from the CPIV3-infected and mock-infected MDBK cells using Transzol UP reagent (Transgen Co. Ltd., Beijing, China) according to the manufacturer’s protocol. Specific primers for amplifying various genes were as follows: for GAPDH mRNA analysis, 5′-GATTGTCAGCAATGCCTCCT-3′ (forward) and 5′-GGTCATA AGTCCCTCCACGA-3′ (reverse) were used; for HSPA5 mRNA analysis, 5′-GTGCCCACCA AGAAGTCTCA-3′ (forward) and 5′-CTTTCGTCAGGGGTCGTTC A-3′ (reverse) were used; for HSP90B1 mRNA analysis, 5′-TCAAGGGTGTTGTGGACTCG-3′ (forward) and 5′-GCT GAAGTGTCTCACGGG AA-3′ (reverse) were used; for HSPA1B mRNA analysis, 5′-AGTC GGACATGAAGCACTGG-3′ (forward) and 5′-TCACCTGCACCTTAGGCTTG-3′ (reverse) were used; and for HSPA6 mRNA analysis, 5′-AGGACAGGCGCAAAGTACAA-3′ (forward) and 5′-TGCTCCAGCTCCCTCTTTTG-3′ (reverse) were used. GAPDH was employed as an internal reference gene. The first-strand cDNA was synthesized via PrimeScript™ RT Master Mix (TaKaRa, Dalian, China). Then qRT-PCR was performed using the SYBR Premix Ex Taq™ II Kit (TaKaRa) on an ABI Step One thermocycler (Applied Biosystems, CA, USA). The relative expression level of each mRNA was calculated by the 2^-ΔΔct^ method. Three independent biological replicates were performed for each gene.

### Western blot analysis

To further verify the variation in the DEPs identified by the proteomic approaches, HSPA1B was selected for western blot analysis. The CPIV3- and mock-infected cells were collected at 24 and 48 hpi. Equivalent amounts of cell lysate from each sample were collected. After measuring the protein concentrations, equivalent amounts of cellular proteins were separated by SDS-PAGE and transferred onto nitrocellulose PVDF membranes (Millipore, USA). The membranes were incubated overnight at 4 °C with primary rabbit polyclonal antibodies of anti-HSPA1B (Biyotime, Shanghai, China). Then the membranes were further incubated for 1 h with horseradish peroxidase-conjugated goat anti-rabbit secondary antibody (BIOSS, Beijing, China). The protein bands were detected using the ECL Detection Kit (Vazyme, Nanjing, China). β-actin protein was used as an internal control.

## Additional files

Additional file 1: **Figure S1.** Information on the detected proteins in CPIV3-infected MDBK cells (JPG 357 kb)

Additional file 2: **Figure S2.** Detected proteins were annotated in the GO database (JPG 1406 kb)

Additional file 3: **Figure S3.** The top 20 pathways annotated by KEGG (JPG 738 kb)

Additional file 4: **Figure S4** and Data Sheet 5. Proteins were annotated based on the KOG (ZIP 669 kb)

Additional file 5: **Figure S6 and S7** Heat map and scatterplot (ZIP 1116 kb)

Additional file 6: **Figure S8.** GO annotations for the up-regulated and down-regulated proteins (JPG 2482 kb)

Additional file 7: Data Sheet 9. The 93 DEPs were annotated into six KEGG pathway categories (XLS 32 kb)

## Abbreviations

COG: Cluster of orthologous groups of proteins
CPE: Cytopathic effect
CPIV3: Caprine parainfluenza virus type 3
DEPs: Differentially expressed proteins
DMEM: Dulbecco’s modified Eagle’s medium
FDR: False discovery rate
GO: Gene ontology
HSPs: Heat shock proteins
iTRAQ: Isobaric tags for relative and absolute quantification
KEGG: Kyoto encyclopedia of genes and genomes
MDBK: Madin-Darby bovine kidney
MOI: Multiplicity of infection
