# Supplementary figures and images for: Proteomics analysis reveals heat shock proteins involved in caprine parainfluenza virus type 3 infection
Source: BMC Vet Res. 2019 May 17;15:151. doi: 10.1186/s12917-019-1897-6 (PMC6525452; doi:10.1186/s12917-019-1897-6)

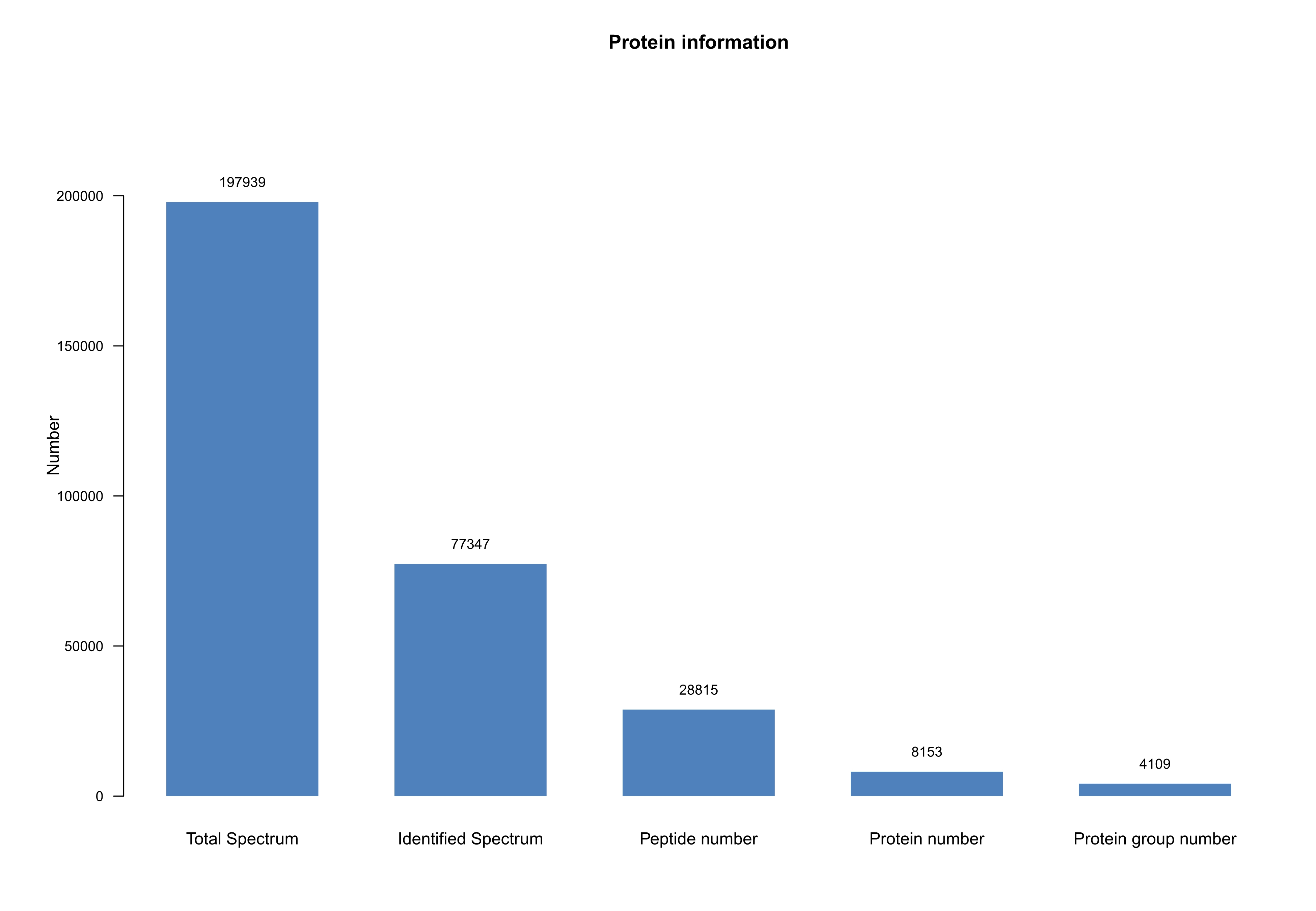

Supplement: Supplementary file 1 — Figure S1. Information on the detected proteins in CPIV3-infected MDBK cells (JPG 357 kb) [file 12917_2019_1897_MOESM1_ESM.jpg]

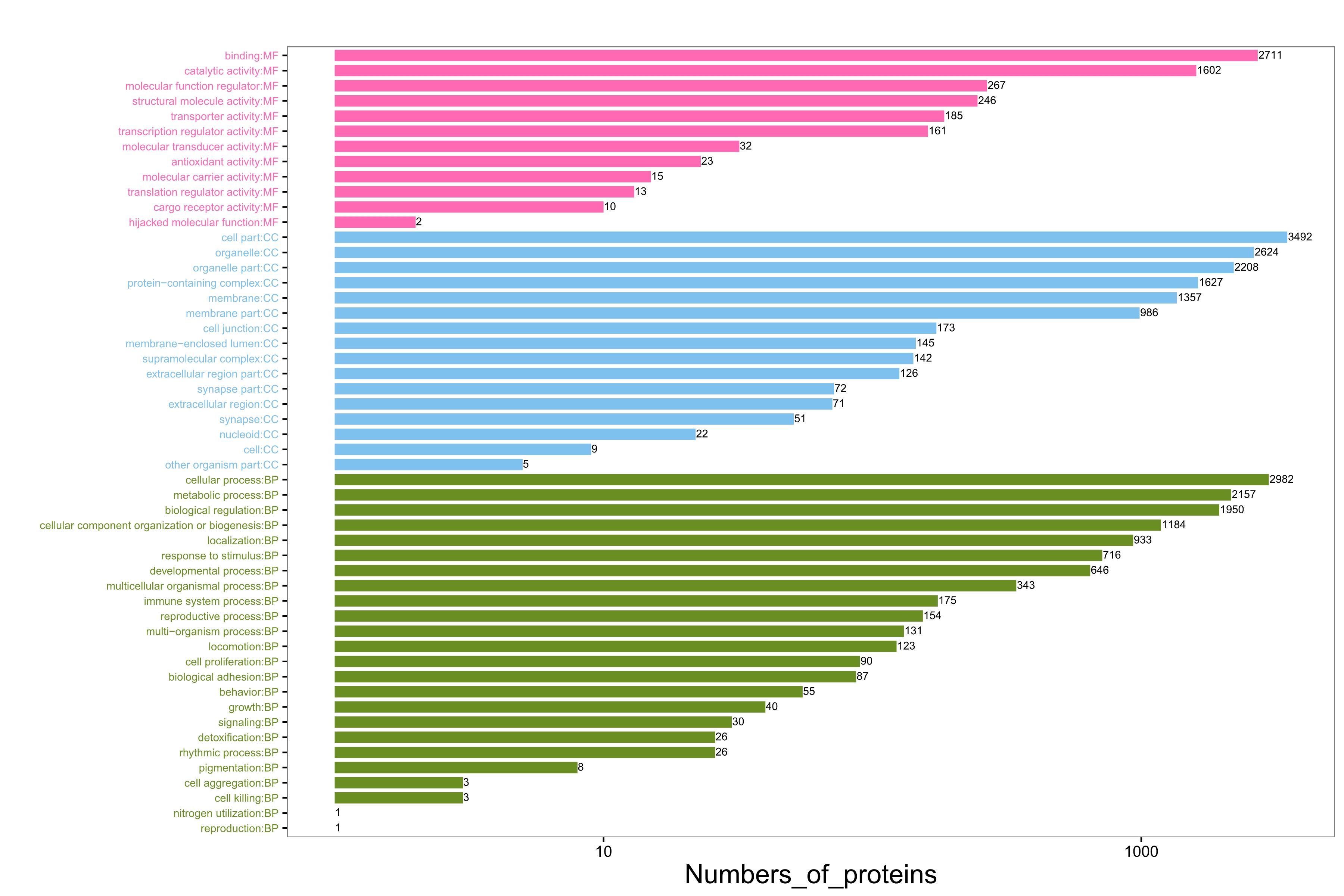

Supplement: Supplementary file 2 — Figure S2. Detected proteins were annotated in the GO database (JPG 1406 kb) [file 12917_2019_1897_MOESM2_ESM.jpg]

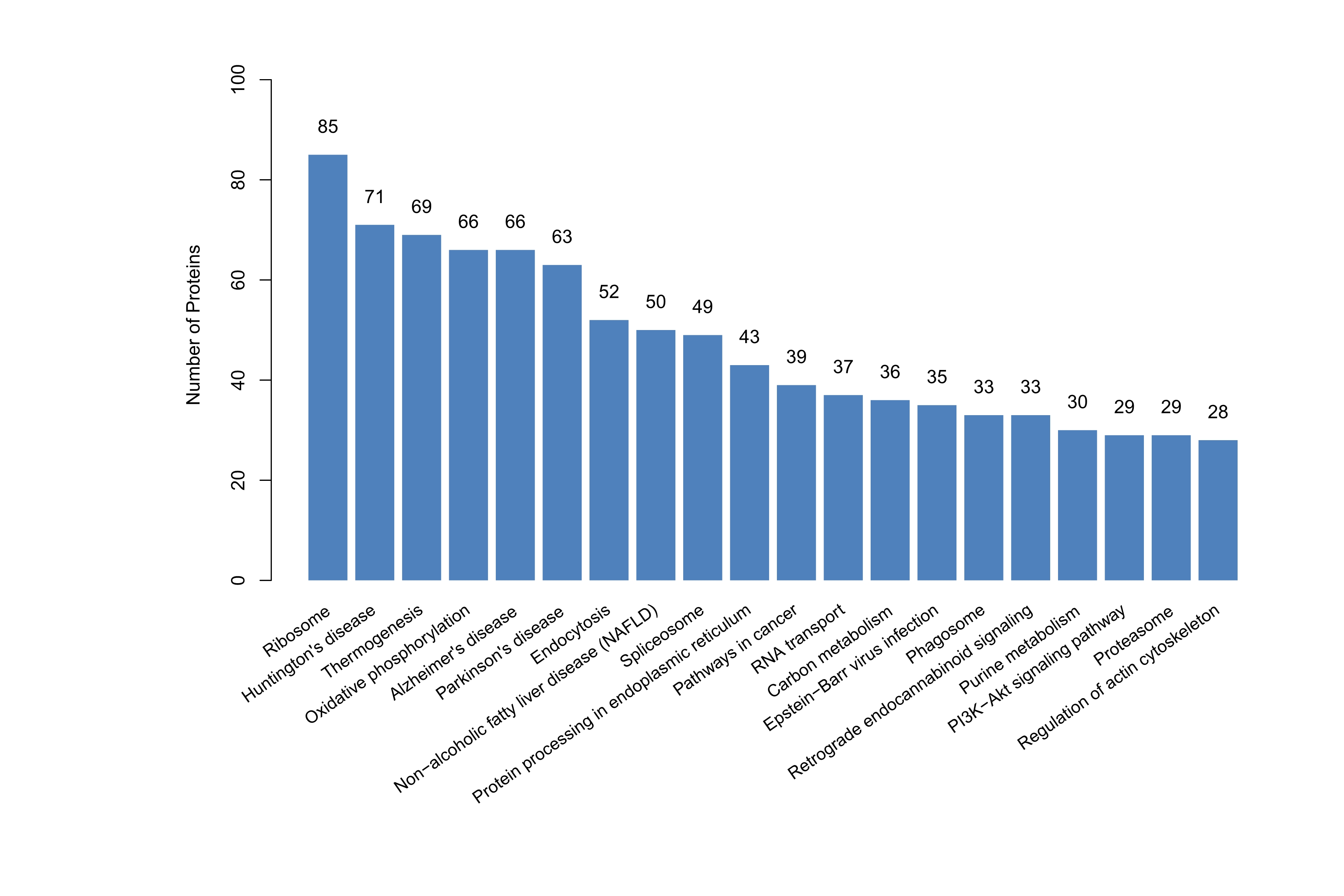

Supplement: Supplementary file 3 — Figure S3. The top 20 pathways annotated by KEGG (JPG 738 kb) [file 12917_2019_1897_MOESM3_ESM.jpg]

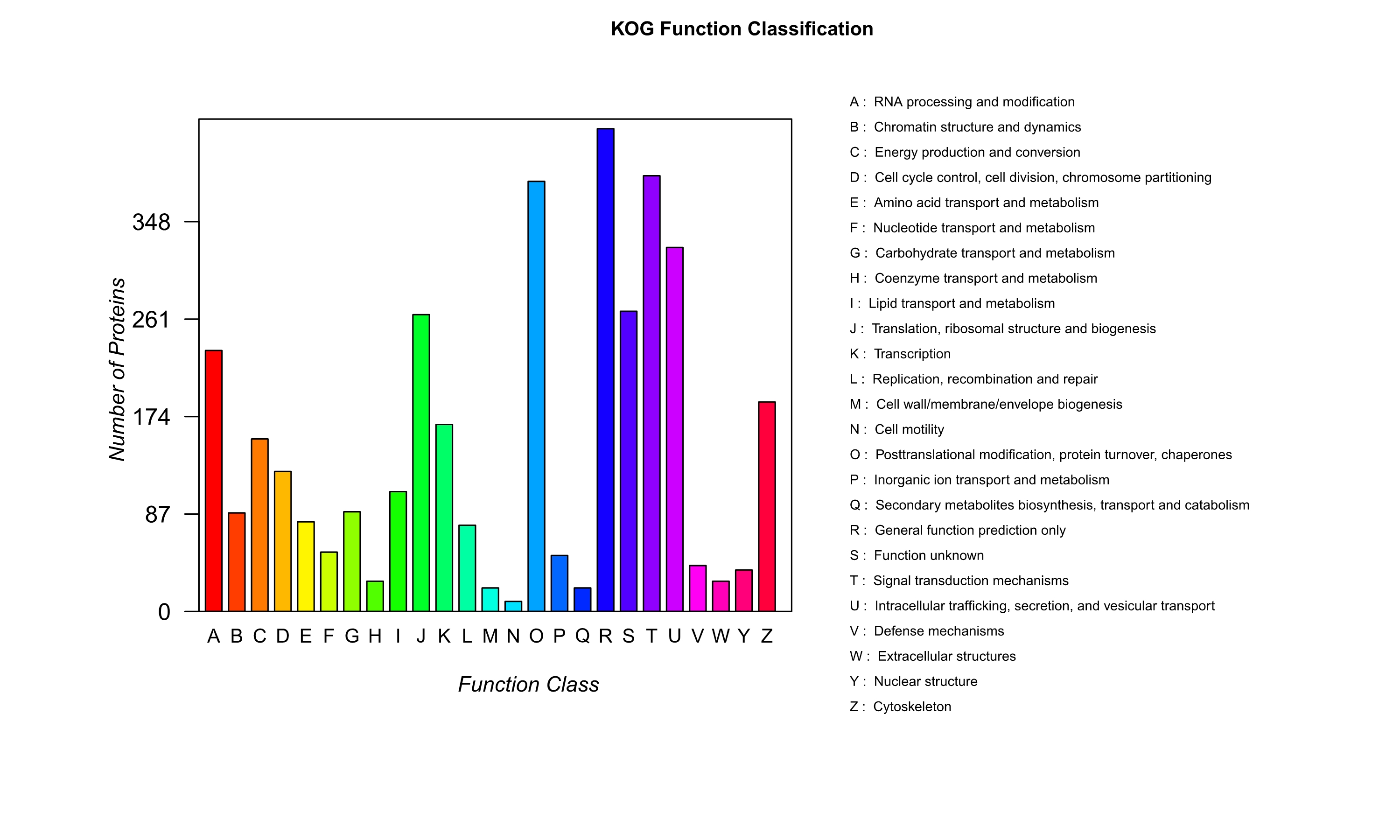

Supplement: Supplementary file 4 — Figure S4 and Data Sheet 5. Proteins were annotated based on the KOG (ZIP 669 kb) [file 12917_2019_1897_MOESM4_ESM.zip › Supplementary figure 4.jpg]

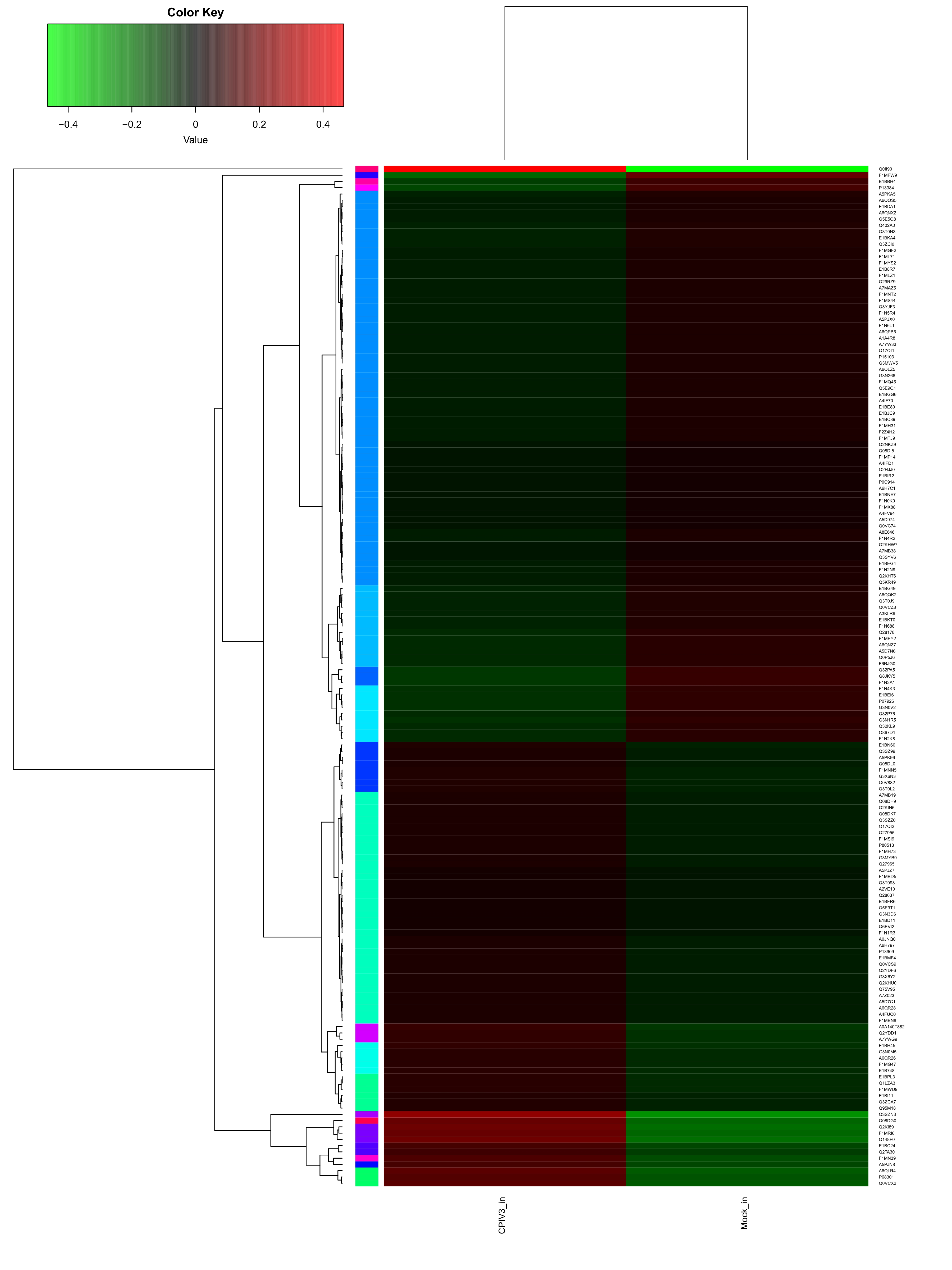

Supplement: Supplementary file 5 — Figure S6 and S7 Heat map and scatterplot (ZIP 1116 kb) [file 12917_2019_1897_MOESM5_ESM.zip › Supplementary figure 6.jpg]

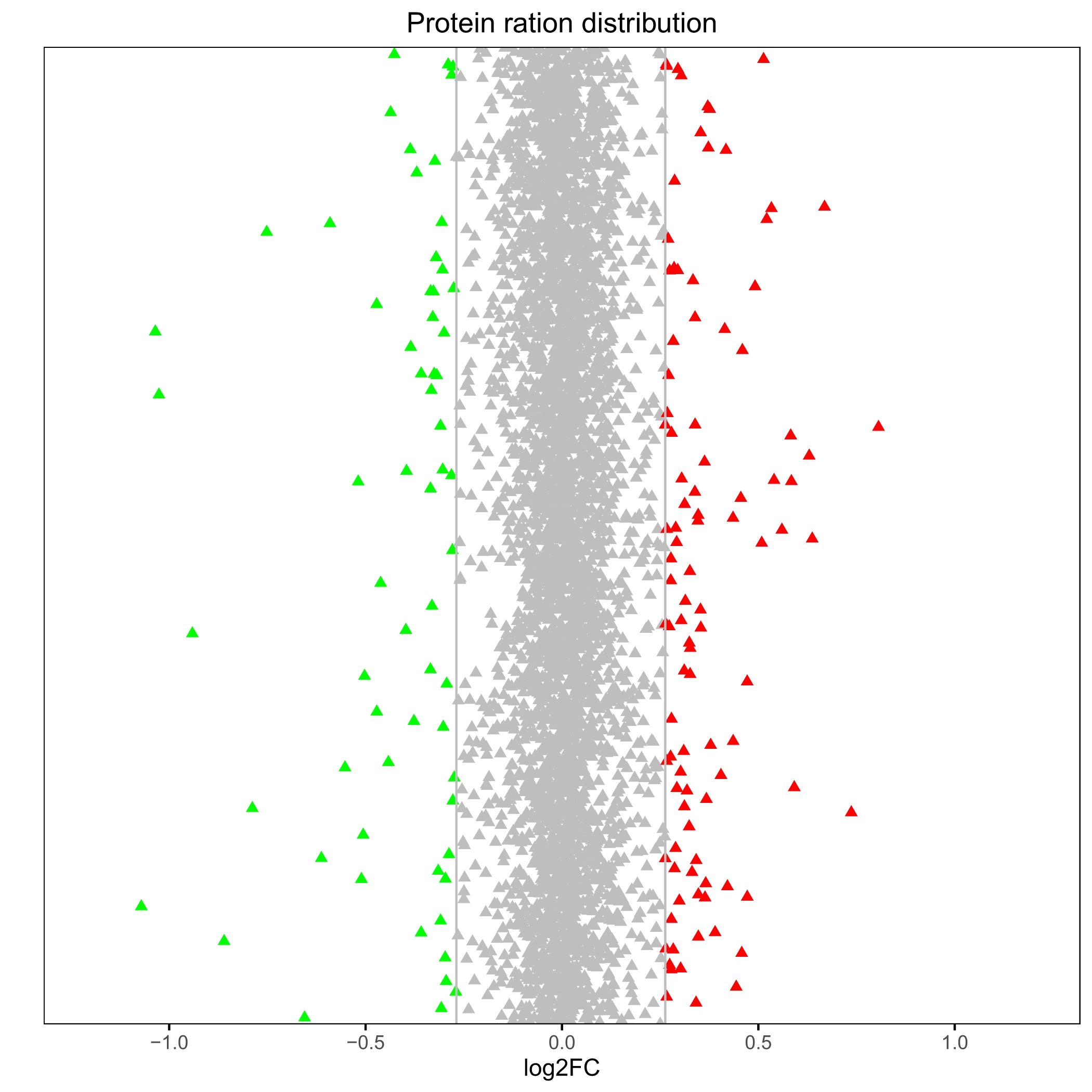

Supplement: Supplementary file 5 — Figure S6 and S7 Heat map and scatterplot (ZIP 1116 kb) [file 12917_2019_1897_MOESM5_ESM.zip › Supplementary figure 7.jpg]

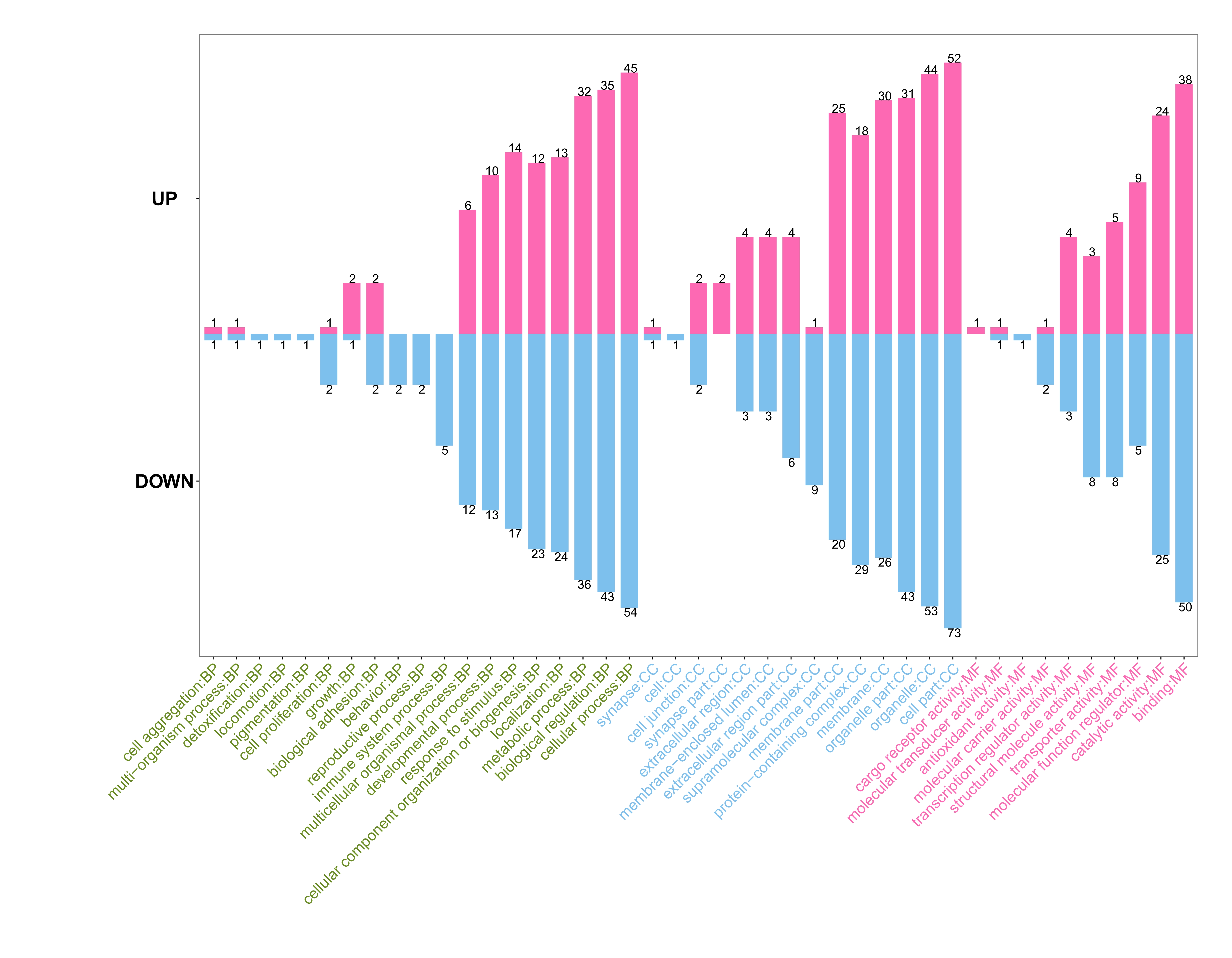

Supplement: Supplementary file 6 — Figure S8. GO annotations for the up-regulated and down-regulated proteins (JPG 2482 kb) [file 12917_2019_1897_MOESM6_ESM.jpg]
